# Modelling the functional genomics of Parkinson’s disease in *Caenorhabditis elegans*: *LRRK2* and beyond

**DOI:** 10.1042/BSR20203672

**Published:** 2021-08-31

**Authors:** Rachael J. Chandler, Susanna Cogo, Patrick A. Lewis, Eva Kevei

**Affiliations:** 1 School of Biological Sciences, University of Reading, Reading, RG6 6AH, U.K.; 2 Department of Biology, University of Padova, Padova, Via Ugo Bassi 58/B, 35121, Italy; 3 Royal Veterinary College, University of London, London, NW1 0TU, U.K.; 4 Department of Neurodegenerative Disease, UCL Queen Square Institute of Neurology, University College London, London, WC1N 3BG, U.K.

**Keywords:** Caenorhabditis elegans, Functional Modelling, GWAS, leucine rich repeat kinase, neurodegeneration, Parkinson’s disease

## Abstract

For decades, Parkinson’s disease (PD) cases have been genetically categorised into familial, when caused by mutations in single genes with a clear inheritance pattern in affected families, or idiopathic, in the absence of an evident monogenic determinant. Recently, genome-wide association studies (GWAS) have revealed how common genetic variability can explain up to 36% of PD heritability and that PD manifestation is often determined by multiple variants at different genetic loci. Thus, one of the current challenges in PD research stands in modelling the complex genetic architecture of this condition and translating this into functional studies. *Caenorhabditis elegans* provide a profound advantage as a reductionist, economical model for PD research, with a short lifecycle, straightforward genome engineering and high conservation of PD relevant neural, cellular and molecular pathways. Functional models of PD genes utilising *C. elegans* show many phenotypes recapitulating pathologies observed in PD. When contrasted with mammalian *in vivo* and *in vitro* models, these are frequently validated, suggesting relevance of *C. elegans* in the development of novel PD functional models. This review will discuss how the nematode *C. elegans* PD models have contributed to the uncovering of molecular and cellular mechanisms of disease, with a focus on the genes most commonly found as causative in familial PD and risk factors in idiopathic PD. Specifically, we will examine the current knowledge on a central player in both familial and idiopathic PD, *Leucine-rich repeat kinase 2 (LRRK2)* and how it connects to multiple PD associated GWAS candidates and Mendelian disease-causing genes.

## Introduction

Parkinson’s disease (PD) is a common, progressive and multi-system neurodegenerative disorder, for which there is currently no disease modifying therapeutic. Affecting 2% of the population over 65 [1], PD is clinically characterised by the development of a progressive resting tremor, bradykinesia and rigidity, responsive to dopamine pathway therapeutics [2–4]. Although predominantly considered a movement disorder, debilitating non-motor symptoms of PD are common in affected individuals and heterogeneous in their presence and extent [2,4–6]. These can include hyposmia, sleep disturbances and autonomic dysfunction, leading to postural hypotension, constipation and urinary incontinence [7]. Psychological conditions such as anxiety and depression have a high incidence throughout PD progression [5–7], along with cognitive impairment, with dementia affecting approximately 60% of individuals 12 years post diagnosis [8]. PD is driven by the degeneration of dopaminergic neurons in the *substantia nigra pars compacta*, leading to the cardinal motor symptoms, and neuropathologically distinguished by the accumulation of Lewy bodies, protein aggregates mainly composed of fibrillar α-synuclein [9]. At the point of PD diagnosis, α-synuclein pathology in the brain is widespread [9], accounting for the diversity and heterogeneity of non-motor symptoms, with an estimated 80% of dopaminergic neurons in the *substantia nigra* lost at the onset of diagnostic motor symptoms [7]. Hence, the development of novel, disease modifying therapeutics to halt or slow the progression of PD is an area of extensive research, underpinned by our understanding of cellular pathways perturbed in PD pathogenesis.

**Figure 1:**
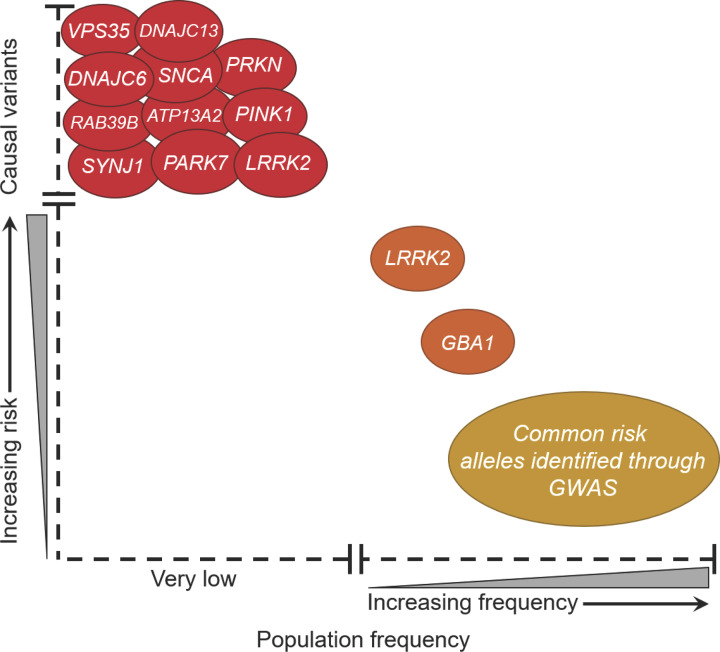
The emerging genetic architecture of Parkinson’s disease Mutations and variants in several genes have been identified to be causal or increase susceptibility to PD. Genes indicated in red, which have a very high or causal link with PD development, show Mendelian inheritance and are rare in the population. Genes indicated in orange confer a greater risk of PD development, but with incomplete penetrance. These genes have been identified through both Mendelian and GWAS studies. The expansion of GWAS targeting idiopathic PD has highlighted a plethora of common alleles, illustrated in yellow. These are very common in the population and confer a heightened susceptibility to PD development. Different PD linked mutations in LRRK2 confer varying risk of PD development and penetrance. There are protective variants in LRRK2, which significantly reduce the risk of PD development, highlighting the central role LRRK2 may play in PD pathogenesis.

The complex aetiology of idiopathic PD is a rapidly expanding area of research, with a plethora of genetic and environmental factors associated with the lifetime risk of PD development [1,10–12]. Approximately 90% of late adulthood-onset PD cases are idiopathic, defined by the absence of an evident monogenic determinant, while the remainder are familial, showing monogenic, Mendelian patterns of inheritance [13]. In most cases, Mendelian PD is symptomatically indistinguishable from the idiopathic disease [13–15], suggesting a potentially shared pathophysiology. PD causative mutations have been identified in a plethora of genes summarised in Figure 1, many of which converge on common pathways including endosomal sorting, vesicle trafficking, lysosomal and mitochondrial function [16]. However, increasing evidence suggests that familial and idiopathic PD can follow an oligogenic pattern of inheritance, in which variants in a number of key genes result in an increased susceptibility to PD onset [17,18]. About 30% of individuals with PD linked to a pathogenic mutation in a Mendelian PD gene also have a concurrent variant in one or more known PD associated genes [18], potentially accounting for variance in disease progression and penetrance between affected individuals. Additionally in post-mortem studies of individuals affected by idiopathic PD with dementia, 23% had more than one genetic variant in known PD associated genes, compared with 10% in an unaffected control population without PD [19]. Importantly, through functional studies of Mendelian disease genes in a diverse range of *in vitro*, *in vivo* and *in silico* models [20,21], substantial progress has been made in elucidating the molecular mechanisms of neurodegeneration in PD.

Common genetic variants identified through genome-wide association studies (GWAS) targeting increased risk of developing idiopathic PD [22] have improved our understanding of the genetic architecture of PD [23–26]. A recent, comprehensive meta-analysis of PD GWASs identified 90 genomic risk signals at 78 genomic loci associated with increased lifetime risk of PD, accounting for up to 36% of heritable risk for PD [24]. Although a great deal of further research is required to conclusively identify the causative variants of these loci, many of the candidate genes converge upon similar cellular pathways shown to be perturbed in Mendelian cases of the disease, notably endocytic vesicle trafficking [16], lysosomal and mitochondrial function [25].

The burden of multiple, common variants within risk loci, which may be present in a single individual, contribute to a polygenic risk score of lifetime PD development [24]. Importantly, a polygenic risk score does not account for environmental factors, which also impact on PD risk and may therefore not be clinically useful in diagnosis, if considered alone [22]. However, when taken in conjunction with prodromal PD symptoms, polygenic risk scores may help in stratifying groups for clinical trials with enhanced genetic precision for targeting, as novel drugs are developed [22]. Identified genes vary in their effect size upon polygenic risk score depending on their functionality, with common variants in known PD associated genes driving larger effect sizes [22,24]. An additional challenge of determining the effect size of novel GWAS loci upon polygenic risk score is that functional modelling for these genes in the context of PD are frequently yet to be undertaken. Thus, our understanding of the mechanism of action of these novel loci and impact upon risk of PD development is incomplete.

The nematode worm, *Caenorhabditis elegans* (*C. elegans*), provides an excellent model system for understanding underlying disease biology in neurodegenerative conditions [20,27,28] and is a promising candidate for functional dissection of the complex genetic architecture of PD. Between 60-80% of human genes have orthologues in *C. elegans* [29] and its genome was the first of any multicellular organism to be completely sequenced [30,31]. Thus, along with the extensive experimental tools available in this system, it facilitates the generation of simple, *in vivo* gene function models. *C. elegans* have been widely used as a model for ageing [32], with their short lifespan and rapid reproduction rate facilitating straightforward and fast study of this process. *C.elegans* are highly amenable to neurobiology, as they have a fully mapped neural connectome [33,34], in which each neuron is characterised in its neurochemistry, connectivity and functionality, often with attributable behavioural phenotypes to each neural circuit to test [35,36]. Furthermore, the majority of neurotransmitters utilised in neuronal signalling in the human brain, are also produced and utilised in the *C. elegans* neuronal system [30,37,38]. Most Mendelian PD genes have orthologues in *C. elegans* (Table 1) and multiple models for PD have been developed. These have demonstrated substantial functional conservation with their human counterparts and yielded important insights into gene function [20,21].

**Table 1:** Mendelian Parkinson’s disease genes and their *C. elegans* orthologues

PD Patient Phenotype	Mendelian PD Gene	Average Age of PD Onset	Number of Confirmed Pathogenic Mutations	Inheritance	GO Biological Functions Disrupted in PD Gene Functional Models	*C. elegans* Orthologue	*C. elegans* Models Reported		
							Deletion	Transgenic Expression	RNAi
Typical PD	***LRRK2***	58	10	Dominant	Endosomal Network, Endocytosis, Vesicle trafficking, Microtubules, Lysosomal Function, Macroautophagy	***lrk-1***	+	+	+
	***VPS35***	50	1		Endosomal Network, Endocytosis, Vesicle trafficking, Lysosomal Function	***vps-35***	+	-	+
	***SNCA***	45	4		Lysosomal stressor	***-***	-	+	-
	***PINK-1***	35	143	Recessive	Mitophagy	***pink-1***	+	-	+
	***PARKIN***	31	14		Mitophagy	***pdr-1***	+	-	+
	***PARK7***	58	5		Oxidative Stress Response	***djr-1.1***	+	-	+
						***djr-1.2***	+	-	-
Parkinsonism	***DNAJC13***	67	0	Dominant	Endosomal Network	***rme-8***	+	-	+
	***DNAJC6***	Juvenile	1	Recessive	Endocytosis	***dnj-25***	+	-	+
	***ATP13A2***	Juvenile	6		Lysosomal Function	***catp-5***	+	-	+
						***catp-6***	-	-	+
						***catp-7***	-	-	-
	***SYNJ1***	Early-onset	3	Recesive	Endosomal Network, Endocytosis	***unc-26***	+	-	+
	***RAB39B***	Early-onset	3	X-linked Recessive	Vesicle Trafficking	***rab-39***	-	-	+

Mendelian PD genes with clinical data regarding PD patient phenotype, mode of inheritance and average age of onset are shown with the corresponding orthologue genes in *C. elegans*. Data have been obtained from Mendelian PD clinical studies [1,14,39–46]. The number of confirmed pathogenic mutations has been obtained from NCBI ClinVar database [47], using the filtered by mis-sense and pathogenic mutations filters. ClinVar database lists 10 pathogenic variants for LRRK2, however, of these only 7 variants have been clearly shown to be pathogenic, suggested by multiple reports from independent kindreds. *Caenorhabditis* *elegans* orthologues have been determined using Ortholist2 search of Mendelian PD genes [29]. Existing *C. elegans* models were obtained from reported phenotype data on each orthologue’s Wormbase entry. GO Biological Functions have been identified utilising G-profiler. Most Mendelian PD genes have an orthologue in *C. elegans*, the gene function of which has been previously studied through either gene deletion, RNAi silencing, transgenic overexpression of the gene or the combination of these methods.

**Figure 2:**
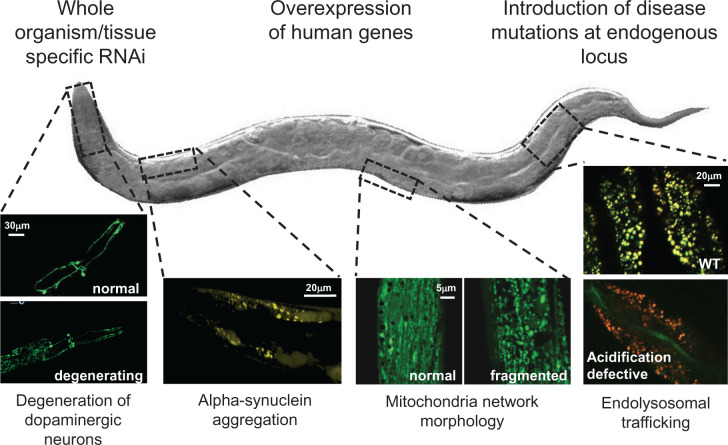
Selected examples of the extensive *C. elegans* toolbox for PD gene functional modelling Multiple genetic approaches can be utilised to investigate gene function in *C. elegans.* PD pathology can be investigated in *C. elegans* through *in vivo* microscopy, using established cellular reporters of dopaminergic neuron degeneration, α-synuclein aggregation, mitochondria network and lysosomal function.

*C. elegans* present a range of PD relevant behavioural and organismal phenotypes to characterise and test, as outputs of gene function, which range from specific dopaminergic neuron attributable behaviours [36,48–50], *in vivo* microscopy [51], to organismal responses to environmental stressors [35], as summarised in Figure 2. Comprehensive and recent reviews of PD relevant phenotypes in *C. elegans* can be found elsewhere [21,35], and open access databases such as WormBase and WormBook contain extensive, up-to-date information on organism genes, *C. elegans* biology and experimental protocols [52,53]. Functional studies of PD in *C. elegans* have, to date, been undertaken using gene deletions, RNAi silencing and transgenic overexpression of the human protein of interest (Table 1 and Figure 2) [20,54,55], but the development of CRISPR/Cas9 has enabled the introduction of specific point mutations in *C. elegans* orthologues [56], for functional modelling from a new perspective.

**Figure 3:**
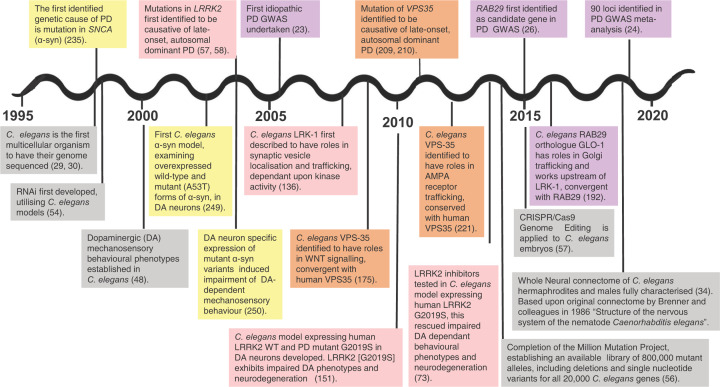
A Quarter Century of advances in human PD genetics, *C. elegans* technologies and functional modelling of Mendelian PD in *C. elegans* Timeline of advancements in our understanding of PD genetics, from the identification of the first mendelian PD gene encoding for α-synuclein in 1997, to the novel and expanding insights recently gleaned through GWAS. Human PD genes are indicated above the timeline and have individual colours assigned to them, with the milestones in *C. elegans* research of this gene or orthologues beneath the timeline indicated with the same colour. Concurrent with the growth of human genetic data in PD, there has been much advancement in *C. elegans* technologies relevant to PD research, illustrated with grey boxes. As a model, *C. elegans* has been pivotal in the development of RNAi technology, whole organism genome sequencing and recently, CRISPR/Cas9 genome editing, enabling the dissection of the effect of point mutations in orthologues. Many of the Mendelian PD genes presented have been studied in *C. elegans* functional models, with promising conservation demonstrated. Some of these are discussed in depth, throughout this review.

In this review, we will evaluate the potential modelling approaches of the functional genetics of PD using *C. elegans*. We angle our focus on the central PD player *LRRK2* and its interplay with Mendelian PD genes such as *VPS35*, which is integral to endosomal function, and the PD hallmark α-synuclein, along with the consistent GWAS locus and potential new therapeutic target, *RAB29*. The concurrent growth of PD genetics and *C. elegans* technologies, summarised in Figure 3, opens an expansive window for the development of simple *in vivo* models, enabling further elucidation of gene function and advancing our understanding of PD pathogenesis.

### The role of LRRK2 in PD and pleiotropy in neurodegenerative disease

**Figure 4:**
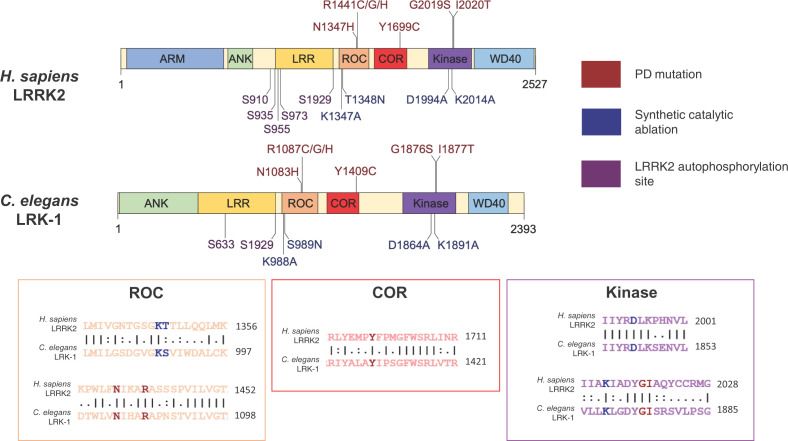
Conservation of domain organisation and amino acid residues between *H. sapiens* LRRK2 and *C. elegans* LRK-1 Human LRRK2 and *C. elegans* LRK-1 share substantial conservation in terms of domain structure, particularly within the catalytic core incorporating the ROC-COR and Kinase domains. The Armadillo domain (ARM), Ankyrin domain (ANK) Leucine Rich Repeats (LRR) and WD40 domain are scaffold regions, facilitating PPIs. Domain information has been obtained from ProSite and Interpro sites, domain visualisation has been generated in DOG1.0 [64]. Sequence alignment has been obtained through analysis of full-length protein sequences with Emboss_001_Needle, pairwise sequence alignment online tool, Matrix: EBLOSUM62, Gap penalty:12, Extend penalty:2 [65–67]. Key residues, that are mutated and pathogenic in PD (labelled with red), and residues, the synthetic mutation of which causes catalytic ablation with demonstrated functional consequences (highlighted in blue) are conserved in *C. elegans*, with high or similar sequence identity in surrounding residues. Two of the five key autophosphorylation sites found in LRRK2 are also present in LRK-1 (shown with purple labels).

Through candidate gene sequencing and recombination mapping in 46 families with autosomal dominant, late-onset PD, seven coding variants in *LRRK2* have been identified to be causative for PD since the first description of mutations in 2004 (Figure 4) [57–60]. Mutations in *LRRK2* are the most common cause of monogenic PD [13], with potential emerging roles of the protein in idiopathic PD [61–63]; therefore, it has been intensively investigated for the development of therapeutics [63].

LRRK2 incorporates a Ras-like GTP-ase domain (Ras of complex proteins), known as ROC, with an adjacent C-terminal of ROC, known as COR domain. These are the defining feature of the ROCO protein family, of which LRRK2 is the most studied member. Adjacent to the ROC-COR is a kinase domain [68–70], as shown in Figure 4. LRRK2 also encompasses scaffold armadillo, ankyrin and the eponymous Leucine Rich Repeat (LRR) domains that facilitate protein–protein interactions (PPIs) at the N-terminus and a WD40 terminal domain at the C-terminus [71]. Notably, PD pathogenic mutations are clustered in the catalytic ROC-COR and kinase domain and lead to enhanced kinase activity [72–77], a key molecular hallmark of *LRRK2* PD [78], which has attracted great attention from a therapeutic perspective (Clinical Trial ID:NCT03710707 [79], NCT04056689 [80]). Several mutations are located in the ROC domain and disrupt GTP hydrolysis [81–83], thereby increasing the proportion of LRRK2 molecules bound to GTP rather than GDP, enhancing auto and substrate phosphorylation [75,83–88]. The interdependence between GTPase and kinase activities is further supported by evidence that synthetically engineered GTP binding ablation mutations in LRRK2 significantly impair kinase activity *in vitro* [89,90], although this is not reflected in all models [91,92]. LRRK2 acts as a homodimer [93,94], suggesting that the enzymatic core functions via an interdependent mechanism, with the ROC domain activating the kinase domain when in its GTP bound state [68,86]. This is contentious, as in alternative, well conserved bacterial models, LRRK2 monomerization occurs following GTP binding, but is found as a dimer when GTP bound or nucleotide free [95]. In addition, LRRK2 is likely in equilibrium between a monomeric, cytosolic and dimeric, membrane bound form [96]. Intriguingly, PD associated mutations in the ROC-COR domain were proposed to alter LRRK2 dimerization and cellular localisation [91,92]; however, the detailed mechanisms as to how the GTPase and kinase domain reciprocally cross regulate activity remains elusive [76,86].

The most common LRRK2 mutation associated with PD, G2019S, is located in the kinase domain and causes a modest 2-fold increase in kinase activity and phosphorylation of substrates *in vitro* [77] and *in vivo* [97], while increasing the LRRK2^G2019S^ autophosphorylation activity 4-fold [98]. The LRRK2^R1441C^ mutation, located in the ROC domain, disrupts GTP hydrolysis, enhancing both substrate phosphorylation and autophosphorylation activity by approximately 3 to 4-fold [89,97,99]. These differences in kinase activity might explain the lifetime risk of PD in *LRRK2* mutation carriers, with higher penetrance observed for LRRK2^R1441C^ carriers [1,10], although with a later age of onset when compared with LRRK2^G2019S^ carriers [1], illustrating that more complex mechanisms may be in action. In addition, while the impact of the LRRK2^G2019S^ substitution is restricted to the kinase pocket of LRRK2, any alteration in the GTPase domain is likely to exert broader biochemical and cellular effects on protein function [90].

The majority of individuals with *LRRK2* PD present with classical PD symptoms and exhibit variable Lewy body pathology upon post mortem examination [10,57]. Interestingly, all *LRRK2* PD cases exhibit significant tau accumulation [57,58,100–102], a pathology observed in some idiopathic PD cases [103], particularly in PD with dementia [104]; however, this is not a diagnostic hallmark of PD upon autopsy [4,9]. Moreover, the tau gene *MAPT* is within a consistent idiopathic PD GWAS locus [24,103], illustrating its potential relevance in idiopathic PD. Lewy bodies and tau have a pleomorphic role in neurodegenerative disease pathology [105,106]. The former are pathognomonic for dementia with Lewy bodies [107], with α-synuclein found in glial cytoplasmic inclusions in Multiple System Atrophy (MSA) [108] and are found as a secondary protein aggregate pathology in up to half of Alzheimer’s disease (AD) cases [109]. Meanwhile, tau is a prominent player in multiple neurodegenerative conditions including AD [110], progressive supranuclear palsy (PSP) [111] and frontotemporal dementia (FTD) [112]; hence, study of LRRK2 with its associated tauopathy and Lewy body aggregation may be applicable to a range of neurodegenerative conditions.

*LRRK2* is notable for exhibiting pleiomorphic pathology in neurodegeneration and in the peripheral immune system [113]. In terms of neurodegeneration, variants in the *LRRK2* locus have recently been identified through GWAS as candidates for increased survival in the tauopathy PSP [114]. Additionally, LRRK2 is highly expressed in many cell types of the immune system [115] and its expression is increased in the B cells, T cells and CD16+ monocytes of idiopathic PD patients, compared with the unaffected control population [116]. This suggests there may be a mechanistic role for LRRK2 in PD relevant inflammation. Notably, variants in *LRRK2* have been observed to increase susceptibility to *Mycobacterium leprae* infection [117], the microbe causative of leprosy, characterised by peripheral neuron damage. Meanwhile, alternative variants in *LRRK2* have been shown to increase the risk of Crohn’s disease [118], an inflammatory condition of the gastrointestinal tract. This further illustrates that studies of LRRK2 may shed additional insights into wider disease pathophysiology. In recent years, LRRK2 kinase hyperactivation has been implicated in idiopathic PD, while *LRRK2* has remained a consistent candidate emerging from idiopathic PD GWAS [22,119–121] suggesting that *LRRK2* functional modelling will provide insights into idiopathic disease, a current challenge in PD research.

### LRRK2 as a therapeutic target

LRRK2 is a promising, druggable target for developing a disease modifying therapeutic for PD, with the potential to be applicable in some cases of idiopathic disease presented with LRRK2 hyperactivation [63]. However, upon the onset of diagnostic PD symptoms, disease pathology is already well established. Concurrent with the development of novel therapeutics, early biomarkers for PD and LRRK2 hyperactivation are under extensive investigation, with the aim to enable targeted pharmacological therapies early in disease pathogenesis or at a prodromal stage [122,123].

For over a decade, it has been known that LRRK2 inhibitors targeting kinase activity can protect against PD associated phenotypes driven by LRRK2 phosphorylation, in *in vitro* and *in vivo* models [124]. Inhibitors have been under extensive development, and in pre-clinical models they have abated α-synuclein mediated neurodegeneration [125,126]. Currently, LRRK2 kinase inhibitors are undergoing early stage clinical trials for safety and efficacy (Clinical Trial ID:NCT03710707 [79], NCT04056689 [80]). Furthermore, splice-switching antisense oligonucleotides (ASOs) targeting *LRRK2* have demonstrated that in human patient-derived cells and murine humanised models, LRRK2 levels can be stably reduced, leading to reduced LRRK2-mediated phosphorylation activity [127]. Reduced α-synuclein inclusions and dopaminergic neuron loss have been observed in the brain of *LRRK2*-ASO treated mice exposed to α-synuclein, while peripheral, off target effects of loss of LRRK2 expression were avoided through direct CNS injection of the ASO [128]. ASOs targeting *LRRK2* are also entering early stage clinical trials (Clinical trial ID:NCT03976349 [119]), with promising potential. Indeed, ASOs are approved for clinical use in the treatment of spinal muscular atrophy in over 40 countries [120], illustrating their relevance in neurodegenerative disease therapeutics.

The development of novel, disease modifying therapeutics for PD hinges upon the thorough understanding of perturbed pathways and the identification of druggable targets that arise, often elucidated through gene functional studies. Substantial progress has been made in utilising *C. elegans* as a simple *in vivo* model for PD genes and as a platform for high-throughput screening of chemical and genetic modifiers [21]; however, there is extensive room for further development and understanding.

### The evolutionary conservation of LRRK2 in *C. elegans*

The ROCO proteins are conserved even in simple eukaryotes, such as the slime mould amoeba *Dictoyostelium discoideum* [121], in which they were first described. Humans have two LRRK paralogs, LRRK1 and 2, while invertebrate models, such as *Drosophila melanogaster* and *C. elegans* have only one, dLRRK2 and LRK-1 respectively. It has been hypothesised that *LRRK2* arose as a gene duplication event, following the protostome-deuterostome split [129,130]. Studies of evolutionary gene duplications have suggested that splits can lead to sub-functionalisation in which the ancestral gene functions are duplicated into two separate genes [131]. In the instance of LRRK2, if sub-functionalisation has occurred, it may have retained similar functions to LRK-1. However, the functional conservation extent between *C. elegans* LRK-1 and LRRK2 is not yet established. Here, we will discuss current models of LRK-1 function, in contrast with established models of LRRK2 function in order to evaluate its suitability as a model for LRRK2 in PD.

Protein sequence alignments between LRK-1 and LRRK2 demonstrate that many key residues mutated in *LRRK2* PD, or synthetically mutated to ablate catalytic activity, are conserved in *C. elegans* LRK-1 [132,133], implicating potential functional conservation between LRK-1 and LRRK2 (Figure 4). PD associated mutation or enzymatic residue conservation is not illustrated between human LRRK2, when aligned with LRRK1, and differential PPI networks of LRRK1 and 2 suggest divergent cellular function [134,135]. In terms of sequence identity, the extent of conservation between LRK-1, LRRK1 and LRRK2 are broadly similar. Between LRK-1 and LRRK1, there is a 20.9% amino acid identity (35.9% similarity), while between LRK-1 and LRRK2, there is a 20.3% identity (36.3% similarity) [65,136]. LRRK1 and LRRK2 phosphorylate distinct subsets of RAB proteins, downstream effectors of LRRK activity [137], and mutations of LRRK1 lead to a rare bone condition, osteosclerotic metaphyseal dysplasia [138–140], also indicative of divergent functionality. Future modelling of key PD pathogenic LRRK2 mutations and variants in *C. elegans* LRK-1 (Figure 4) may shed light on the extent of functional conservation between *C. elegans* LRK-1 and mammalian LRRK1 and LRRK2.

**Figure 5:**
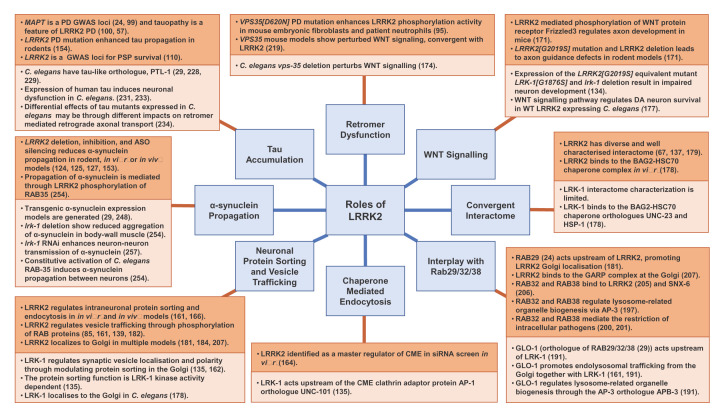
Functional roles of LRRK2, contrasted with *C. elegans* LRK-1 The summary of key pathways and functions implicated in LRRK2 PD (shown in the light blue boxes). Underlying studies describing LRRK2 functions in patient studies, mammalian, and *in vitro* models are shown for each function in dark orange boxes. The relevant *C. elegans* pathways are detailed in the light orange boxes. These demonstrate a promising functional conservation between *C. elegans* LRK-1 and mammalian LRRK2, augmenting LRK-1's relevance for further functional modelling in PD. Further details of all examples detailed here are discussed throughout the main body of text.

When contrasted with LRRK2 mammalian *in vitro* and *in vivo* model systems, functional studies of *C. elegans* LRK-1 suggest it is a promising candidate as a LRRK2 model, as extensively detailed in Figure 5 and throughout this review. Here, we will first discuss the role of LRK-1 deletion and humanised LRRK2 transgenic models, followed by insights gleaned from study of LRK-1. This will be succeeded by the *C. elegans* conservation of the RAB29/LRRK2 axis, retromer dysfunction and protein aggregation, pertinently tau and α-synuclein in modelling LRRK2-linked Parkinson’s pathogenesis, with potential implications for modelling the candidate genes of novel GWAS loci.

### The relevance of LRK-1 deletion models for PD

Studies of *LRRK2* function in *C. elegans* have often been limited to *lrk-1* gene deletion, or transgenic overexpression of human LRRK2 on a wild-type nematode background [132,133,141–143]. Deletion models are useful in understanding physiological LRK-1 function, however in the context of understanding the biology of *LRRK2* mutation driven PD, the modelling needs to be extended to the individual gene variants with proven, or possible pathogenicity. PD-linked mutations in *LRRK2* have consistently been shown to act through a toxic gain of function mechanism, in which phosphorylation activity of LRRK2 is increased [78]. Human genetic studies have illustrated that there are individuals with heterozygous loss of function variants in *LRRK2* [144], leading to an approximately 50% reduction in LRRK2 protein levels [144,145]. These individuals do not present with PD and have no significant health complications [145]. This suggests that in humans there could be compensatory mechanisms for LRRK2 function reduction, and that drug delivered kinase inhibition, or application of ASOs in therapeutics may not be deleterious. However, in pre-clinical rodent and non-human primate animal models, LRRK2 inhibitor treatment presented on-target side effects in the lung, showing abnormal accumulation of lysosomal related organelles known as lamellar bodies [146]. Encouragingly, this phenotype was reversible [146], and early stage clinical trials of LRRK2 inhibitors have so far showed safety and tolerability [79,80]. Pre-clinical *in vitro* models illustrate that LRRK2 inhibition does not significantly affect LRRK1 phosphorylation activity, supporting their distinct structure and function [147]. In the context of *C. elegans*, deletion mutant and RNAi loss of function *lrk-1* models would not provide an orthologous tool to modelling the PD gain of function *LRRK2* mutations. Furthermore, *C. elegans lrk-1* deletion models do not show impaired dopaminergic phenotypes and behave similarly to wild-type [141], supporting the human genetics findings [145].

### Insights from transgenic expression of LRRK2 in *C. elegans*

Using humanised *C. elegans* models, through overexpressing human LRRK2 in wild-type and PD mutant form, it is possible to model gain of function mutation effects [76]. However, caution is needed when interpreting data, as little is known about the background phosphorylation activity and potential interplay, dimerization and functional redundancy of wild-type *C. elegans* LRK-1, when human LRRK2 is co-expressed. Adult *C. elegans* with a wildtype *lrk-1* background, expressing LRRK2^G2019S^ and LRRK2^R1441C^ in the dopaminergic neurons, alongside a dopaminergic neuron specific GFP reporter, exhibit significantly reduced GFP fluorescence by day 3 of adulthood, indicative of dopaminergic neurodegeneration [148]. This has been replicated in further studies, illustrating that these models have a robust, PD associated phenotype, relevant to *LRRK2* modelling [76,149]. Often, rodent models do not recapitulate key PD hallmarks in terms of neuropathology [150]; however, viral-mediated PD mutant LRRK2 overexpression induces dopaminergic neuron loss [151,152]. The development of PD relevant phenotypes and neuropathology in rodents occurs over several months, while *C. elegans* have an advantage in speed, sample size and exhibition of robust phenotypes within days.

Furthermore, *C. elegans* are highly amenable to environmental toxin assays and drug screens to evaluate the interplay with transgenic expression of human proteins of interest [153,154]. *C. elegans* strains overexpressing the PD associated LRRK2^G2019S^ and LRRK2^R1441C^ mutant proteins in neurons show reduced survival in response to oxidative stress induced by the environmental toxins rotenone and paraquat, widely used in toxin-based models of PD [155], when compared with animals expressing wild-type LRRK2 [142,148]. However, these studies did not examine whether protection was kinase dependent, through LRRK2 inhibition, or expression of kinase inactive LRRK2. Notably, *C. elegans* overexpressing wildtype LRRK2 show enhanced survival to oxidative stress and increased lifespan compared with non-transgenic wild-type animals. This suggests a LRRK2-driven protective effect, while deletion, or RNAi silencing of *lrk-1* in non-transgenic lines have shown a significantly reduced survival under these conditions [148,156]. Overexpression of LRK-1 is yet to be utilised in oxidative stress assay, so it is currently unknown whether this would have a similar effect to heterologous expression of LRRK2. These results suggests that PD mutant LRRK2 has a reduced efficacy in oxidative stress protection in *C. elegans*. However, the expression of these pathogenic LRRK2 variants in wildtype *C. elegans* background provides enhanced stress resistance compared with wild-type, non-transgenic *C. elegans*. This suggests that examination of the role of endogenous LRK-1 mutant proteins, with physiological expression levels, might provide better understanding of how these mutations impact survival of the organism, or the dopaminergic neurons. Nevertheless, this illustrates LRK-1s global role in oxidative stress survival, which may not be neuronal specific or restricted to neurons.

Additionally, the transgenic lines discussed above have been utilised as *in vivo* models to test early LRRK2 inhibitors LRRK2in1 and TTT-3002 [157,158]. Dopaminergic behavioural phenotypes and neurodegeneration observed in vehicle treated nematodes expressing LRRK2^G2019S^ or LRRK2^R1441C^ [76] are rescued following treatment with any of the two LRRK2 inhibitors [76]. However, the effect of LRRK2 inhibitors upon endogenous LRK-1 is unknown, and molecular readouts for LRK-1 phosphorylation activity remain to be identified. Similar effects in nematode behaviour, dopaminergic neurodegeneration and LRRK2 phosphorylation activity were demonstrated in a recent study utilising allosteric inhibition of LRRK2 kinase activity by vitamin B12 in LRRK2^G2019S^ expressing lines [149], in support of the robust, relevant phenotypes and promise of *C. elegans* in pharmacological assays.

The use of transgenic human LRRK2 expressing *C. elegans* models have contributed to our understanding of *LRRK2* function, while studies of LRK-1 suggest it may share functional conservation with LRRK2. Further characterisation and understanding of *C. elegans* LRK-1 function may shed further insight into LRRK2 mediated pathology from a novel model angle.

### LRRK2 and *C. elegans* LRK-1 functionality in vesicle trafficking, endocytosis and WNT signalling

Data obtained upon transgenic expression of *C. elegans* LRK-1 generally supports the hypothesis that it may share some functional conservation with LRRK2. In 2007 *C. elegans* LRK-1 was suggested to have a role in regulating synaptic vesicle localisation to the dendrites of neurons, through modulating protein sorting in the Golgi in a kinase activity dependent manner [133], while deletion of LRK-1 leads to increased sensitivity to tunicamycin induced endoplasmic reticulum stress [132]. Accordingly, enrichment at Golgi and the regulation of intraneuronal protein sorting through this network has also been reported in studies of LRRK2 in rat cortical neuron cultures *in vitro*, and in *Drosophila melanogaster* and rodent models, *in vivo* [159]. Expression of *C. elegans* LRK-1^K1726A^ and LRK-1^I1877T^ mutant proteins in the *C. elegans lrk-1* deletion mutant, (equivalent missense point mutations of the synthetic kinase ablation LRRK2^K2014A^ and PD associated kinase hyperactive LRRK2^I2020T^, respectively) has demonstrated that kinase activity of LRK-1 is crucial to its function in determining polarised vesicle localisation in axons of *C. elegans* sensory neurons, suggesting shared conservation in enzymatic activity and functionality between LRK-1 and LRRK2 [133,160]. LRRK2 kinase activity has recently been shown to be essential in modulating axonal transport, with abnormal activation of kinesin *in vitro* and *in vivo* in PD LRRK2^G2019S^ mutant models [161]. Interestingly, in the 2007 study of LRK-1, localisation of synaptic vesicle proteins was shown to depend on the *C. elegans* protein UNC-101, an orthologue of the clathrin adaptor protein AP-1 [160] involved in vesicle trafficking from the Golgi to endosome. Clathrin adaptor proteins are integral to vesicle transport processes, including clathrin-mediated endocytosis (CME), a process also regulated by LRRK2 [162]. Alterations to the CME process have been recognised in PD [163–165] and other neurodegenerative conditions [166,167], implicating potential functional overlap in the *C. elegans* model.

WNT/β-catenin signalling is an essential pathway in dopaminergic neurogenesis during development and also contributes to synapse formation and neuroprotection during ageing [168]. As PD is caused by the loss of dopaminergic neurons, the WNT/β-catenin pathway is of great interest in the development of disease modifying therapeutics targeting neurogenesis [168]. Importantly, LRRK2 plays key roles in the WNT signalling pathways [78,169–171]. LRRK2 modulates axonal development through inducing phosphorylation of the WNT receptor Frizzled3, a component of the planar cell polarity (PCP) pathway in mice [169], with both loss of function and kinase overactive LRRK2 mutants presenting axon guidance defects. Likewise, in *C. elegans* the WNT signalling pathways modulate neuronal development [172–174]. Interestingly, expression of the *C. elegans* LRK-1^G1876S^ (the equivalent of human pathogenic LRRK2^G2019S^) in wild-type, or *lrk-1* deletion mutant background, revealed significant defects in canal-associated neuron development [132]. Furthermore, in an RNAi screen targeting endogenous genes in wildtype *C. elegans* lines overexpressing human LRRK2^G2019S^, endogenous nematode WNT signalling pathway components and axon guidance genes had the largest effect sizes in determining dopaminergic neuron survival [175]. Thus, this shared phenotype of impaired neuronal development and survival through WNT pathways between transgenic *C. elegans* LRK-1 and vertebrate LRRK2 models further strengthens the conserved roles of LRK-1 and LRRK2.

### The shared interactome of LRRK2 and *C. elegans* LRK-1

Comparative study of LRK-1 and LRRK2 interactomes further supports functional conservation and it might provide another strategy to identify candidate pathways as novel therapeutic targets [71]. LRRK2 has been shown to bind the BAG2-HSC70 chaperone complex, acting as a chaperone for LRRK2 [176]. In *C. elegans*, UNC-23 and HSP-1, the orthologues of BAG2 and HSC70 respectively, interacts with LRK-1, highlighting existing conservation of protein interactions [176]. Further functional studies have illustrated that UNC-23 mediates Golgi localisation of LRK-1 in co-operation with HSP-1, and *unc-23* deletion mutants phenocopy the defects in synaptic vesicle localisation [176], as seen in *lrk-1* deletion mutants [132,133,176]. This suggests that UNC-23 and LRK-1 function together in synaptic vesicle localisation.

LRRK2 has an extensively characterised interactome; a stringent search through PINOT, a resource for obtaining quality controlled PPIs in humans and *C. elegans* [177], indicated 1440 interactor hits for LRRK2, replicated with multiple studies and methods (July 2021). In contrast, the LRK-1 interactome is poorly characterised, with only few interactors noted. Due to the existing conservation of interactions and of the molecular and cellular function between LRK-1 and LRRK2, *C. elegans* could provide an excellent tool to drive interactome-based functional studies. These could be investigated in the context of the ageing organism, including environmental and genetic PD risk factors, utilising the high-throughput nature of this model to assess the efficacy of genetic and chemical disease modifiers.

## The role of Parkinson’s disease GWAS candidate risk gene *RAB29* in *LRRK2* and idiopathic PD

RAB proteins are a superfamily of small GTPases, with diverse regulatory roles in vesicle formation, trafficking and endosomal transport. A subset of these RABs are key effector substrates of LRRK2 phosphorylation [178]. *RAB29*, also known as *RAB7L1*, has been a consistent PD GWAS hit [24] and the RAB29 protein has been shown to act upstream of LRRK2 [179], while RAB3, RAB5, RAB8, RAB10, RAB12, RAB35 and RAB43 have been demonstrated to be downstream effector substrates regulated by LRRK2 phosphorylation [180]. *RAB29* has been stratified through unbiased LRRK2 PPI arrays as a candidate risk gene, which may bridge the gap between familial *LRRK2* and idiopathic PD [159,179,181]. Thus, our understanding of the mechanisms of both *LRRK2* and idiopathic PD may be furthered by functional studies of *RAB29*.

RAB29 has been demonstrated to be a selective master regulator of LRRK2, acting upstream and leading to LRRK2 kinase recruitment, localisation and activation on the Golgi membranes, via binding of RAB29 to LRRK2s ankyrin repeat domain [179,182,183], with resultant increased LRRK2 kinase activity leading to changes in the trans-Golgi network morphology [184]. However, in another study *RAB29* knockout had no impact on basal LRRK2 phosphorylation activity [185], suggesting a potentially more complex mechanism in LRRK2 modulation and PD pathogenesis. Furthermore, RAB29 is also a substrate of LRRK2 in human HEK293 cells; LRRK2 with kinase overactive PD mutations LRRK2^R1441C^, LRRK2^Y1699C^ and LRRK2^G2019S^ present a 4-fold increase of LRRK2 mediated phosphorylation of RAB29 [178], suggesting interdependent regulation between these two proteins. Other key substrates of LRRK2 are RAB8A and RAB10, with RAB8A shown to accumulate at the trans-Golgi network following RAB29 mediated LRRK2 recruitment [182]. Furthermore, upon stress induced lysosomal enlargement, LRRK2 is targeted to the lysosomal membranes via RAB29 [183]. This leads to an accumulation of RAB8A and RAB10, which attenuates the enlargement of lysosomes, essential for maintaining their integrity and function [183]. Thus, RAB29 is integral to LRRK2 biology and function, further highlighting the need to develop our understanding of the pathways in a range of models.

### The conservation between RAB29 and *C. elegans* orthologue, GLO-1

*C. elegans* has an orthologue for *RAB29*, *glo-1*, which shows conservation in its function in the endolysosomal transport pathway [186]. A coding variant RAB29^K157R^ has been detected in an idiopathic PD patient [187], however its pathogenicity, as well as its mechanism of action is unconfirmed. This variant position is conserved in *C. elegans* GLO-1 and is located within the G5 loop, involved in binding of small G-proteins to nucleotides [71,188]. Importantly, *C. elegans* GLO-1 has been shown to act upstream of *C. elegans* LRK-1 [189], congruent with studies of human LRRK2, further suggesting it may be relevant for further functional modelling of RAB29. GLO-1 is also the *C. elegans* orthologue for the closely related human RAB32 and RAB38, and it is most similar to these proteins in terms of protein sequence identity [29]. GLO-1 shares 45.9% and 46.1% sequence identity with RAB32 and RAB38 respectively, while with RAB29 it shares a 40.7% identity [65,136]. Functional studies of GLO-1 have demonstrated that it shares conservation with all three RAB proteins to varying extents. *In vivo* model organisms do not always show exact orthologue conservation with the human gene of interest. For example, mouse models display significant differences in biology and biochemistry of LRRK2 when compared with human [150]. Despite this, much useful information has been gleaned through functional modelling of LRRK2 in mice [128,151,152], which can be contrasted or supported by consistent findings in alternate *in vivo*, *in vitro* and *in silico* models, along with findings reported in studies of PD patients. Thus, *C. elegans* GLO-1, when taken in conjunction with findings in other models, will be a useful model for further understanding RAB29 function, along with shedding insight into its close counterparts RAB32 and RAB38.

GLO-1 may not just be a potential functional model with relevance to PD, as *RAB29*, *RAB32* and *RAB38* have been highlighted as genes of interest in various neurodegenerative conditions. Functional studies of *RAB29* in the presence of *C9orf72* hexanucleotide repeat [190], causative of amyotrophic lateral sclerosis with FTD (ALS-FTD), have illustrated its role in vesicle trafficking [191]. Additionally, *RAB38* has been identified as a significant GWAS hit in a behavioural variant subtype of FTD [192], while *RAB32* has been associated with ER stress and mitochondrial dysfunction in multiple sclerosis [193]. This illustrates that this small sub-group of RABs may play an important role in the maintenance of neuronal health and an *in vivo* reductionist model of their functions focusing on GLO-1 may be applicable to further research areas.

### The LRK-1/GLO-1 interplay in *C. elegans*

Congruent with the human RAB29/LRRK2 axis, in *C. elegans* both endogenous LRK-1 and neuronally expressed human LRRK2 on a *lrk-1* deletion background have been shown to act downstream from GLO-1 in axon termination [189]. Notably, key residues of RAB29, routinely utilised to synthetically ablate or constitutively activate its GTPase function *in vitro* [190], are conserved in *C. elegans* GLO-1. On the other hand, key LRRK2 phosphorylation target sites T71 and S72 of RAB29 [194] are not conserved. GLO-1 and LRK-1/LRRK2 converge to regulate axonal morphology, highlighting their importance in the nematode central nervous system, and like RAB29 and LRRK2, they have lysosomal roles [189]. In motor neuron axons, GLO-1 and LRK-1 act to modulate endo-lysosomal trafficking or endo-lysosomal maturation, suggesting similar functions to their human counterparts [189]. Transgenic expression of PD mutant LRRK2^G2019S^ or LRRK2^R1441C^ in *lrk-1* deletion mutants efficiently supresses the axonal abnormality phenotype exhibited in *lrk-1* or *glo-1* deletion mutants [189], suggesting high functional conservation. However, the effect of human RAB29/32/38 expression in *glo-1* deletion mutant background was not investigated, nor was the Golgi localisation of both proteins [189]. Furthermore, the effect or presence of LRK-1/LRRK2 mediated phosphorylation of GLO-1 was not investigated [189], as robust phosphorylation readouts have not been developed for PD relevant orthologues in *C. elegans*. This highlights the need for additional studies to further assess the LRK-1/GLO-1 mechanistic conservation with the comparatively better understood LRRK2/RAB29 interplay.

### The relevance of RAB32 and RAB38 in understanding RAB29 and LRRK2 function

Functional studies of human *RAB32* and *RAB38* implicate some shared pathways with *RAB29*, augmenting the relevance of *glo-1* for further study. RAB32 and RAB38 have been demonstrated to act co-operatively to regulate lysosomal biogenesis and modulation of lysosome-related organelles (LROs) [195]. In *C. elegans*, GLO-1 modulates the biogenesis of LROs, which are intracellular compartments for storage, sharing some characteristics but coexistent with conventional lysosomes [186,196]. Furthermore, human RAB32 and RAB38 co-ordinate lysosomal biogenesis through the clathrin adaptor protein AP-3 [195], which is a known effector substrate of LRRK2, acting downstream from RAB29 [189]. Likewise, studies of GLO-1 have identified modulation of LRO biogenesis through APB-3, the *C. elegans* orthologue of AP-3, via *C. elegans* LRK-1 [197], mirroring the human pathway [189]. Additionally, nematodes with deletion of *glo-1* or *apb-3*, or double deletion mutants of these genes, show decreased LRO biogenesis [189], suggesting convergent biology and importance in this function, as shared in the RAB32 and RAB38 interplay with AP-3. These mutual processes, pathways and interactors suggest that GLO-1 shares high functional conservation with RAB32 and RAB38.

Similarly to RAB29, RAB32 and RAB38 also share convergent biology with LRRK2, although their mechanisms of interaction may differ. RAB32 and RAB38 have been shown to mediate phagosome restriction of intracellular pathogens, notably *Mycobacterium leprae*, the causative agent of leprosy [198]. Interestingly, single-nucleotide polymorphisms (SNPs) in *LRRK2* have been identified through GWAS as predisposing towards leprosy [117], and a polymorphism in *RAB32* [199] has also been associated with disease susceptibility, implicating potential shared pathways in the immune response between LRRK2 and RAB32 and RAB38. It is currently unclear whether these immune pathways also contribute to PD pathogenesis [200]. However, it has been hypothesised that PD associated mutations in *LRRK2*, prevalent in multiple human populations [201], may confer greater immunity to selected infectious diseases. This may have led to a balanced selection of *LRRK2* PD mutations, resulting in antagonistic pleiotropy, with potential advantageous roles of mutation in immunity, but increased risk of PD development in later life [113,202].

LRRK2 physically interacts with RAB32 and RAB38 through its armadillo domain [203], convergent with the LRK-1 and GLO-1 interaction [189]. Furthermore, RAB32 and RAB38 bind to sortin-nexin 6 (SNX-6), a transient subunit of the retromer complex, affecting retromer dependent Golgi trafficking [204]. Recent studies have demonstrated that LRRK2, bound to trans-Golgi localised RAB29, interacts with the GARP complex, stabilising syntaxin-6 and promoting retrograde transport to the trans-Golgi network in a kinase dependent manner [205]. Importantly, syntaxin-6 and all GARP subunits are conserved in *C. elegans* and RNAi knockdown of GARP subunits in *C. elegans* expressing human LRRK2^G2019S^ in the dopaminergic neurons [141] induces dopaminergic neurodegeneration [205]. This proposed functional convergence of RAB29, RAB32 and RAB38 at the trans-Golgi network, with influences on trafficking and LRRK2 interaction suggest that GLO-1 may have retained similar functions of its three evolved human orthologues and may therefore be useful in gaining further mechanistic insight into the events linking these proteins to neurodegeneration.

## Convergence of LRRK2 with VPS35 linked Mendelian Parkinson’s disease

A mutation in Vacuolar Protein Sorting 35, VPS35^D620N^, was identified in 2011 in a small number of families with late onset, autosomal dominant PD, showing similar symptomatic presentation to idiopathic PD [206–208]. VPS35 is an integral subunit of the retromer complex, implicated in retrograde transport between the endosomes and the trans-Golgi network [209]. VPS35 and its link with the retromer was fist described in the unicellular *Saccharomyces cerevisiae* [209–211]. *C. elegans* has a well conserved, direct *VPS35* orthologue, *vps-35* [212]. Interestingly, VPS35^D620N^ has been shown to enhance LRRK2 kinase activity 6-fold *in vitro*, in CRISPR engineered murine fibroblasts and neutrophils isolated from PD patients carrying VPS35^D620N^ [99]. This suggests an interplay between LRRK2 and the retromer in PD, a plausible hypothesis for the molecular mechanism of *VPS35^D620N^* PD, further highlighting the roles of LRRK2 and the endosomal network in neurodegeneration.

Studies conducted in *C. elegans* thus far have demonstrated that VPS-35 and the retromer have a role in modulating WNT signalling, determining neuronal migration during development [172,173,213–215]. The role of *VPS35* in WNT signalling is conserved in other invertebrate models, such as, but also in mammals as observed in cell culture studies [215,216], suggesting maintained functional conservation through evolution. Recently, heterozygous VPS35^D620N^ mouse models have been reported to display WNT/ß-catenin signalling dysfunction [217]. The potential pathogenic perturbation of the WNT signalling pathway [217], converging with its impairments illustrated in the *LRRK2* PD models [78], implicates WNT signalling as a core process with relevance to PD pathogenesis. In *C. elegans*, VPS-35 has been implicated in trafficking of the nematode orthologue of the excitatory AMPA glutamate receptor, GLR-1. Deletion of *vps-35* leads to a significant reduction in the number of GLR-1 puncta on the post-synaptic surface of ventral nervous cord dendrites and suggests a role for the retromer in GLR-1 recycling [218]. Coherently, in iPSC neuronal cultures derived from people with *VPS35^D620N^* PD, APMA receptor trafficking was dysregulated and receptors were mislocalised [219]. This shared role between *C. elegans* VPS-35 and its human orthologue suggest retained functional conservation through evolution.

### The role of retromer dysfunction in tauopathies

Similarly to *LRRK2, VPS35* has been associated with tauopathies in *in vivo* and *in vitro* modelling [220,221]. It is currently unclear whether individuals with *VPS35^D620N^* PD exhibit typical α-synuclein pathology, as due to the rarity of mutations in this gene autopsy studies to date have been very limited [222]. In 2008, a post mortem study was undertaken with one individual from a Swiss family with multi-generational PD, who was later identified as a VPS35^D620N^ carrier [8]. Key PD relevant brain areas were not analysed, but α-synuclein pathology was not observed [8]. Hence, additional studies to obtain further insight into α-synuclein and tau pathology are required. *In vivo* murine models with VPS35^D620N^ endogenously knocked in exhibit progressive neurodegeneration driven by tauopathy [220], while retromer has been shown to modulate the lysosomal clearance of tau *in vitro* [221]. The evidence of VPS35^D620N^ resulting in enhanced LRRK2 kinase activity [99] further suggests that tau pathology could be present in *VPS35^D620N^* PD, as tauopathy is a hallmark of *LRRK2* PD [102]. Additionally, kinase overactive LRRK2^G2019S^ has been demonstrated to enhance neuronal transmission of tau in mouse models [152]. Tau, a key protein in AD and FTD, is a PD GWAS locus and has been observed in approximately 50% of PD patients post-mortem, illustrating the relevance of functional study of LRRK2 for further mechanistic insights potentially applicable to a range of neurodegenerative conditions.

Like tauopathies, retromer dysfunction has been implicated in multiple neurodegenerative conditions, suggesting potential common perturbed pathways and an essential role in neuronal function. In AD autopsy studies, microarrays have illustrated that retromer is depleted in the hippocampus, within the dentate gyrus and entorhinal cortex, a region in which AD pathology is initiated [223]. Further mechanistic studies have shown that retromer facilitates the trafficking of amyloid precursor protein (APP) through sortilin binding within the retromer, thus retromer deficiency leads to APP accumulation and cleavage to pathogenic ß-amyloid [224], a driver of AD pathogenesis [225]. Thus, further study of the retromer in the well-conserved *C. elegans* model may shed further insight into multiple pathologies.

### Modelling tauopathies in* C. elegans*

*C. elegans* have a tau-like orthologue, *ptl-1* [226,227] and multiple evidence suggests that this protein may cover essential roles in maintaining neuronal integrity through the lifespan when expressed at basal levels [228]. Loss of *ptl-1* is not rescued by expression of human tau isoforms [228], suggesting potentially limited functional conservation between *ptl-1* and human tau. Multiple studies have used transgenic expression of human tau isoforms, investigating the impact of overexpression of wildtype or pathogenic mutant tau in *C. elegans*. Overexpression of wild-type tau in *C. elegans*, or neuronal expression of A152T mutant tau [229], a risk factor for FTD, PSP and atypical tauopathies [112,230] have been demonstrated to induce neuronal dysfunction [231]. Expression of tau^A152T^ in *C. elegans* leads to impaired associative memory, compared with animals expressing wildtype tau [232], and this may be explained by differential impacts on retrograde axonal transport [232], of which the retromer is a central component. The emergence of tau as a contributor to *LRRK2* and *VPS35* linked PD, the emerging aetiological overlap with the tauopathies and the existence of ready to use tauopathy models in *C. elegans*, provides further opportunities for functional studies and modifier screens of tau pathology, with impacts on multiple neurodegenerative conditions.

## Modelling α-synuclein pathology in *C. elegans* to dissect PD gene function

The first gene to be directly implicated in inherited PD was the *SNCA* gene on chromosome 4 [233]. In 1997, a missense coding variant in *SNCA* (A53T) was found to segregate with PD and dementia in a large family of Greek and Italian origin, the Contursi kindred [233]. Soon after, α-synuclein, the protein product of *SNCA*, was identified as the major constituent of Lewy bodies [166]. The aggregation propensity of α-synuclein and its involvement in neurodegenerative diseases had already been reported, albeit without being recognised as α-synuclein, as it had previously been associated with the non-amyloid component of plaques in AD patients [234], who frequently develop Lewy bodies [109,235]. Since 1997, six *SNCA* missense mutations, as well as gene duplications and triplications have been linked to dominantly inherited PD and dementia, suggesting that an increase not only in the aggregation tendency but also in the expression level of α-synuclein can induce toxicity [236]. More recently, the advent of GWAS revealed that the *SNCA* locus is among the major determinants of PD predisposition in the general population [237], with variability in common risk loci contributing to the polygenic risk of lifetime PD development [16,24,107,238,239]. These genetic findings have driven functional research, supported by the evidence that *SNCA*, as many others, is a pleiotropic PD gene and that PD follows an oligogenic pattern of inheritance [18,240]. A key unanswered question therefore is, to what extent interplay between α-synuclein and other PD proteins contribute to the aetiology of PD [241–245].

*C. elegans* do not possess an orthologue of *SNCA*; however, multiple *C. elegans* models have been generated expressing wild-type or mutant human *SNCA* as a transgene, in muscles, neurons or dopaminergic neurons [246,247]. These have been illustrated to recapitulate PD hallmarks of dopaminergic neurodegeneration [155,246,247] and have been employed to study a diverse range of PD relevant pathways, shedding insights into PD pathology. These studies have been discussed in depth in a recent comprehensive review, by Gaeta, Caldwell and Caldwell [248], therefore they will not be extensively covered here. RNAi screens, utilising α-synuclein overexpressing *C. elegans* models, have demonstrated that multiple PD relevant genes, such as orthologues of *PINK-1, PARKIN, ATP13A2* and *DJ-1*, identified in Mendelian familial studies are modifiers of α-synuclein pathology [249]. This suggests promising pathway conservation, illustrating the great potential to dissect the mechanisms of PD relevant genes, in conjunction with transgenically expressed α-synuclein in *C. elegans*.

### The interplay of LRRK2/LRK-1 in α-synuclein transgenic models

Many pathways linking α-synuclein and LRRK2 have been suggested, including cytoskeletal dynamics, ER/Golgi transport, mitochondrial homeostasis and functionality of the degradative systems, leading to the hypothesis that the use of LRRK2 inhibitors might be beneficial in the treatment of synuclein pathology [250,251]. Lack or inhibition of LRRK2 kinase activity or LRRK2 deletion can mitigate neurodegeneration observed in rats after transduction of human α-synuclein via adeno-associated viral vectors (AAVs) [125,126,252]. Indeed, AAVs, together with lentiviral vectors, have been extensively exploited to deliver wild-type and mutant human α-synuclein to the *substantia nigra* of rodents and primates, where they lead to Lewy body formation and neurodegeneration. Conversely, overexpression of the wild-type or mutant human α-synuclein in rodents does not correlate with pathological aggregate formation and degeneration of dopaminergic neurons in the *substantia nigra* [253]. However, expression of human α-synuclein the invertebrate models *D. melanogaster* and *C. elegans* recapitulates the major PD hallmarks such as impaired dopaminergic behaviour and neurodegeneration despite the lack of an orthologue [179,244].

Similar studies of α-synuclein propagation with regard to LRRK2 have been undertaken *in vivo* in rodent models and *in vitro* human derived SH-SY5Y cultures, and contrasted with *C. elegans* LRK-1 [252]. Interestingly, *lrk-1* deletion mutant *C. elegans* shows reduced aggregation of α-synuclein, expressed in the muscle [252]. In the same study, this phenotype was recapitulated in the brain of *LRRK2* knockout rats, injected with AAV vectors of recombinant human α-synuclein. These animals showed significantly reduced number of axons immunoreactive for α-synuclein at 12 weeks [252], consistent with LRRK2 inhibitor studies [125,126]. In a concurrent *in vitro* human SH-SY5Y culture study, this phenotype was determined as kinase activity dependent, with LRRK2^G2019S^ enhancing α-synuclein propagation, through RAB35 phosphorylation, a mechanism demonstrated to be conserved in *C. elegans* [252]. Expression of a constitutively active RAB35 in the *lrk-1* deletion mutant reversed the phenotype of reduced α-synuclein aggregation [252], further illustrating that key mechanisms and pathways in PD pathogenesis are readily modelled in *C. elegans*, despite the simplicity and evolutionary differences of the system.

However, deletion models of PD orthologues, when used in conjunction with α-synuclein expression still need to be approached with caution. A study developing a *C. elegans* model for neuron-neuron propagation of α-synuclein *in vivo* demonstrated that RNAi silencing of PD orthologue genes, including *lrk-1, pdr-1, pink-1, vps-35*, resulted in increased α-synuclein propagation between neurons [254]. Furthermore, more complex mechanisms may be at play, as *lrk-1* expression at the mRNA level has been shown to significantly increase in nematodes expressing α-synuclein in the muscle in the presence of the apoptosis inducer wedelolactone [255] and the flavonoid tambulin [256]. Both of these have been demonstrated to reduce α-synuclein aggregation in *C. elegans* muscles. Dysregulation of *LRRK2* mRNA expression has been described in post-mortem studies of individuals with idiopathic and LRRK2^G2019S^ PD [257]. However, these convergences and inconsistencies in *C. elegans*, with regards to the α-synuclein/LRRK2 interplay, further highlights the need to develop more precise genetic models of *LRRK2* pathology in *C. elegans*, rather than depending solely on deletion and RNAi silencing studies.

## LRRK2 and beyond: The potential of *C. elegans* for functional modelling of Parkinson’s disease GWAS candidate genes

**Figure 6:**
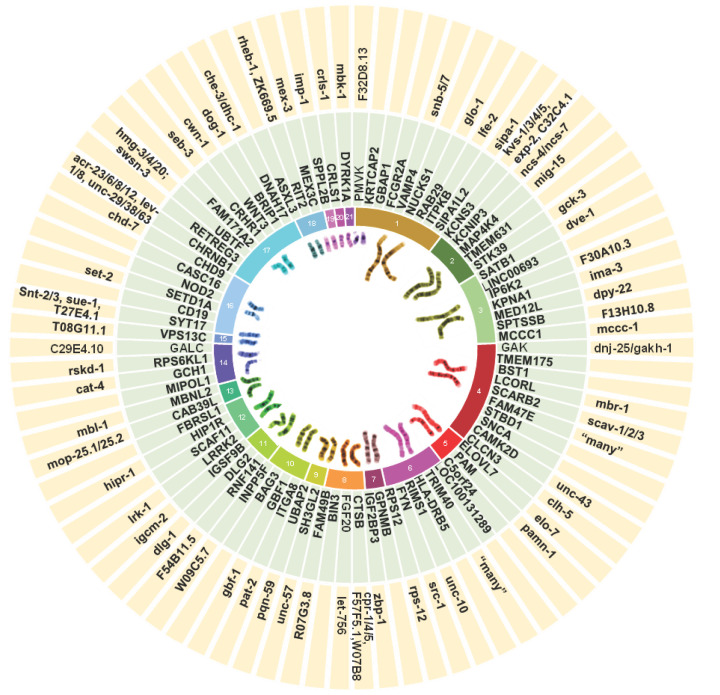
The potential for functional modelling PD GWAS loci in *C. elegans* The most recent PD GWAS meta-analysis by Nalls et al [24] highlighted 90 genome-wide significant risk signals at 78 genomic regions, implicated in idiopathic PD. Search of the *C. elegans* orthologues of the nearest, or most relevant candidate genes suggested by the IPDGC GWAS locus browser [240], using Ortholist2 [29], demonstrated that 64 of the listed candidate risk genes have orthologues in *C. elegans*, yet to be characterised in the context of PD modelling.

The past two decades have witnessed a seismic change in our understanding of the genetic contribution to the aetiology of PD, culminating most recently in the 2019 meta-analysis of PD GWAS, identifying 90 risk loci for PD, that account for up to 36% of PD heritability [24]. In Ortholist2, a database mapping human genes to their *C. elegans* orthologues [29], 64 of the identified genes have direct orthologues in *C. elegans*, detailed in Figure 6. Considering the increasing speed in dissecting the genetic component of PD and the requirement for rapid and robust functional validation, new models are now required to introduce multiple mutations in a single background and study their interplay. Such a scenario is difficult to achieve in rodents, while *C. elegans* possess the optimal feasibility in terms of genetic manipulation, for the generation of oligogenic models, along with RNAi and chemical modulator screening to investigate the impact of genetic and environmental factors. Genome-wide RNAi screens are powerful, but could miss many potential modifiers, with subtle impairments that occur only in specific conditions. Thus, combined risk screens might constitute a novel era in PD research, for which a simple functional model is needed.

As extensively detailed in this review, *C. elegans* orthologues of Mendelian PD genes such as *lrk-1* and *vps-35* show promising functional conservation with their human counterparts *LRRK2* and *VPS35*, when contrasted with a diverse range of *in vitro* and *in vivo* models. It is clear that in many model systems, *LRRK2* is a key player in PD pathway disruption and may further bridge the gap in our understanding of Mendelian to idiopathic PD. GWAS risk genes, such as *RAB29* (*glo-1* in *C. elegans*), also show great promise for further functional modelling in *C. elegans*. This opens the possibility of developing new nematode models to further understand the functionality of novel candidate genes, rapidly emerging through the availability of vast genetic data. The development of CRISPR/Cas9 technologies has enabled the rapid generation of knockin point mutations in *C. elegans* orthologues of human genes [56,258], although this approach is not yet as widely used as deletion, RNAi silencing and transgenic expression of PD-relevant genes [20,55,143,259,260]. Endogenous CRISPR/Cas9 engineered mutations enables precise modelling of human genetic variants that might pose as risk for developing PD [261]. As *C. elegans* is one of the easiest and cheapest multicellular eukaryotic organism to apply precise genome editing to, the choice of this simple nematode for studying PD biology provides fast, cheap *in vivo* modelling with great translational potential [21,29,51], as discussed throughout this review. *C. elegans* functional studies of other Mendelian PD genes and their orthologues, including *PINK1, PARKIN, ATP13A2* and *DJ-1*, or PD risk genes, such as *GBA1*, have proven the strength of this invertebrate model, highlighting highly conserved functions of these genes and proteins in cellular pathways disrupted in PD, including mitophagy, lysosomal degradation and α-synuclein pathology [49,132,175,262–270]. Complex functional interaction of all of these PD proteins and LRRK2 have been described in PD patient samples and in animal models, which supports the idea that LRRK2 is a master regulator of cellular trafficking and quality control pathways, maintaining a cross-talk of a multitude of cellular processes and reflects on the high complexity of Parkinson’s disease pathology [13,271–274]. Developing oligogenic *C. elegans* models, replicating the human genetic changes and evaluating cross-talk of various cellular pathways in PD, will help in enabling the future development of novel, disease modifying therapeutics.

## Open Access

Open access for this article was enabled by the participation of University of Reading in an all-inclusive *Read & Publish* pilot with Portland Press and the Biochemical Society under a transformative agreement with JISC.

## Abbreviations

APP: amyloid precursor protein
ASO: antisense oligonucleotide
CME: clathrin-mediated endocytosis
PD: Parkinson’s disease
PPI: protein–protein interaction
